# The Therapeutic Potential of Cannabidiol in Revolutionising Opioid Use Disorder Management

**DOI:** 10.7759/cureus.50634

**Published:** 2023-12-16

**Authors:** Kelvin Le, Joanne Au, Jean Hua, Khang Duy Ricky Le

**Affiliations:** 1 Melbourne Medical School, The University of Melbourne, Melbourne, AUS; 2 Department of Anaesthesia & Pain Management, The Royal Melbourne Hospital, Melbourne, AUS; 3 Department of Pharmacy, The Royal Melbourne Hospital, Melbourne, AUS; 4 Faculty of Pharmacy and Pharmaceutical Sciences, Monash University, Parkville, AUS; 5 Geelong Clinical School, Deakin University, Geelong, AUS; 6 Department of General Surgical Specialties, The Royal Melbourne Hospital, Melbourne, AUS; 7 Department of Surgical Oncology, Peter MacCallum Cancer Centre, Melbourne, AUS; 8 Department of Medical Education, Melbourne Medical School, The University of Melbourne, Melbourne, AUS

**Keywords:** cannabidiol, opioid replacement therapy, opioid use disorder, opioids, medical marijuana, cannabinoids

## Abstract

Opioid use disorder (OUD) is a significant cause of morbidity and mortality worldwide and is linked to a complex interplay of biopsychosocial factors as well as the increasing overprescription and availability of opioid medications. Current OUD management relies on the controlled provision of opioid medications, such as methadone or buprenorphine, known as opioid replacement therapy. There is variable evidence regarding the long-term efficacy of these medications in improving the management of OUD, thereby necessitating an exploration into innovative approaches to complement, or even take the place of, existing treatment paradigms. Cannabidiol (CBD), a non-psychoactive compound derived from the cannabis plant, has garnered attention for its diverse pharmacological properties, including anti-inflammatory, analgesic, and anxiolytic effects. Preliminary studies suggest that CBD may target opioid withdrawal pathways that make CBD a potential therapeutic option for OUD. This narrative review synthesises current literature surrounding OUD and offers a nuanced review of the current and future role of CBD in managing this condition. In doing so, we highlight the potential avenues to explore with respect to CBD research for the guidance and development of further research opportunities, framework and policy development, and clinical considerations before medicinal CBD can be integrated into evidence-based clinical guidelines.

## Introduction and background

Opioid use disorder (OUD) is a significant cause of morbidity and mortality, globally affecting over 16 million people and responsible for more than 100,000 deaths per year [1-3]. Key reasons for this include overprescription and accessibility to opioid analgesia with undercurrents of other biopsychosocial aetiological factors also at play [4,5]. Often referred to as the opioid crisis or opioid epidemic, OUD remains a significant public health issue [3]. Opioid replacement therapy (ORT) using opioid receptor agonists, such as methadone and buprenorphine, remains the gold standard approach for managing withdrawal symptoms and cravings associated with OUD. Current guidelines stipulate the prescription of ORT based on clinical features indicating opioid abuse, such as somatic withdrawal symptoms of diaphoresis, tachycardia, anxiety and behavioural symptoms of drug-seeking practices, and tunnel vision [3,5]. ORT has shown significant positive outcomes in managing OUD, such as reducing risky behaviour, crime rates, illicit opioid use, and all-cause mortality [6-8].

Despite these benefits, significant limitations associated with current ORT still exist, including the development of dependence and addiction to opioid agonists, risk of overdose, lack of equitable accessibility, and the potential for drug diversion [7,9-11]. These limitations stem from the inherent propensity of these drugs to elicit dependence and addiction, prompting the need to investigate non-opioid options in managing OUD [12,13]. The use of cannabidiol (CBD) has garnered clinical interest in tackling the opioid crisis recently. CBD is a potential non-opioid therapeutic for managing OUD due to its ability to interact with various neurochemical pathways associated with reducing addiction and withdrawal syndromes [14]. Additionally, the proposed analgesic effects of CBD have been suggested to reduce prescription opioid use, which is a significant factor towards opioid accessibility, misuse, and eventual OUD [4,15].

This review aims to provide insight into the potential role of CBD in ameliorating the opioid crisis and its role in the management of opioid dependence disorders. Herein, we will explore the current understanding of OUD and the factors that contribute to this condition and review the present landscape of ORT. The therapeutic potential of CBD in this arena along with its limitations and direction of future research will be further discussed.

## Review

Understanding opioid use disorder

Opioids are a large drug class derived from opium alkaloids found in the resin of opium poppy seeds (*Papaver somniferum*) [16]. Opioids can be naturally derived (morphine, codeine), semi-synthetic or synthetic (oxycodone, hydrocodone), or illegally manufactured (heroin) [3]. In practice, opioids are commonly prescribed as strong analgesics for the management of both acute and chronic pain [16,17]. This occurs through the ability of opioids to bind a variety of opioid receptors (delta, kappa, mu) found in the peripheral and central nervous system. Activation of these receptors triggers opioid signalling pathways that elicit downstream analgesic effects for effective pain management [18].

Despite these positive therapeutic effects, it is important to recognise that opioids are simultaneously considered substances of abuse with a high potential for addiction and misuse that can easily transition into an OUD [18]. Despite their role in modern medicine, opioids are potential substances of abuse due to their addictive potential that can easily spiral into an OUD [18]. OUD describes the repeated, hazardous patterns of opioid misuse that result in the development of tolerance, dependence, and addiction. Its significant worldwide mortality rate arises as a result of respiratory depression from narcosis; studies have highlighted the risk of mortality from individuals with OUD is between six and 20 times higher than that of the general population [1,3]. Opioid dependence results in morbidity that arises from the physiological and psychological symptoms of withdrawal. Physiological symptoms may include myalgia, arthralgia, nausea, vomiting, diarrhoea, and insomnia [19]. Psychological symptoms include compulsive opioid use habits and drug-seeking behaviour, preoccupation with a drug-seeking behaviour, development of drug-related cues and reward salience, an inability to control intake, and emotional and mental instability without opioid use such as anxiety [20-23]. These put a strain on interpersonal relationships, contribute to mounting medical expenses, decrease employability, and place the individual at an increased risk of crime and incarceration [3,24].

Neurophysiological Factors

Chronic opioid use gives rise to neurophysiological maladaptations, resulting in the development of opioid dependence and addiction. These changes are exhibited by the central nervous system effects of the mu-opioid receptor [18]. The activated mu-opioid receptor acts centrally to increase dopamine neurotransmitter release in areas of the brain, including the ventral tegmental area, nucleus accumbens, and dorsal striatum, eliciting a reward phenomenon [5,8,25]. Opioid misuse therefore subsequently leads to intense feelings of euphoria, fuelling the motivation for ongoing opioids that subsequently progresses to dependence syndromes [8]. However, chronic opioid abuse leads to long-lasting neuroplastic adaptations from repeated activation of dopamine activity, resulting in goal-directed behaviour and habit formation [23,26]. These neuroplastic adaptations are responsible for the development of opioid dependence and addiction, resulting in intense withdrawal symptoms with cessation [5,25]. Genetic factors influencing the mu-opioid receptor pathway can also increase the likelihood of developing OUD [3]. Additionally, chronic opioid use results in neurocircuitry changes, such as decreased receptor sensitivity and expression, impaired coupling of receptor activation, dysfunctional intracellular signalling activation, and adaptations in cell signalling pathways, further contributing to the development of opioid tolerance, dependence, and addiction [17,18].

Although conventional understanding and current gold-standard treatments for OUD focus on changes at the mu-opioid receptor level, it is important to note that other key neurophysiological signalling processes are implicated in OUD development. One mechanism is through the dysregulation of the endocannabinoid system through potentiated dopamine signalling with opioid abuse, which can exacerbate OUD [27]. Specifically, current studies have posited that disrupted mesolimbic dopaminergic pathways increase CB1R activity, which consequently potentiates a dopaminergic response in the ventral tegmental area, nucleus accumbens, and dorsal striatum to produce rewarding euphoric effects important in the development of OUD [23,27]. Furthermore, it is interesting to note that CB1R and mu-opioid receptors are colocalised, which may result in reciprocal interactions that further potentiate dopaminergic effects and complicate OUD [23]. Moreover, serotoninergic pathways are disrupted with opioid abuse. Interestingly, differing neurophysiological effects have been documented depending on the nature of opioid pharmacokinetics. Acute opioid use has been demonstrated to result in surges of serotonin (5-HT) release within specific regions of the brain; however, chronic or sustained opioid use contrarily results in a reduced or absent 5-HT response. This impairment of physiological serotoninergic signalling is hypothesised to impair central pain and reward regulation, which can further complicate opioid abuse into OUD [28,29].

Psychosocial Factors

There are several psychological and social factors that increase one’s susceptibility to OUD and the eventual development of opiate dependence and addiction. Mental health disorders and unstable emotional environments increase the likelihood for an individual to misuse opioids [3]. Individuals with a personal history of depression, anxiety, relationship strain, sexual or physical abuse or trauma, and comorbid psychiatric disorders are linked with opioid abuse as a form of self-medication to relieve oneself from aversive events [1,3]. This repeated behaviour eventually forms a learned association between stressful situations and opioid misuse, thereby developing subconscious associations between opioid use, the euphoric effects of opioid abuse and relief from aversive life stressors [30]. Opioid misuse is further compounded by the overprescription and oversupply of prescription-only opioids, with a vast majority of opioid misuse stemming from what was initially a non-medical indication [18]. In Australia, approximately 11% of individuals aged 14 or over were reported to have used opioids for illicit or non-medical purposes, the majority of exposure from pharmaceutical opioids (9.7%) secondary to excessive accessibility from over-prescribing [4,31]. Concerningly, a lack of education about appropriate opioid stewardship practices is a key determinant preceding OUD and oftentimes inappropriate continuation of prescription opioids after completion of pain management regimes can result in persistent opioid misuse and progression into OUD [1,5].

OUD is a chronic, relapsing disease due to the addictive properties of opioids and the burdensome nature of withdrawal symptoms [1]. One study found that approximately 60% of abstainers succumbed after three months, with 75-85% relapsing after 12 months [17]. A general framework of OUD and relapse can be conceptualised as an ‘addiction cycle’, consisting of a negative withdrawal symptom experience, followed by a phase of preoccupation, anticipation, and craving, finally culminating in binging and intoxication [20]. Relapse of abstinence can be triggered by various factors, including uncontrollable withdrawal symptoms, incentive salience from opioid overprescription, exposure to opioids, and relief from aversive life stress [30,32]. Although a vulnerability to relapse will be lifelong, maintaining abstinence for at least five years has been shown to substantially reduce the likelihood of relapse [1]. Current ways of managing OUD include psychosocial treatment (including cognitive behavioural therapy), ORT, or medically supervised opioid cessation (detoxification) with symptomatic management [3].

The current landscape of opioid replacement therapy

Although there are several current strategies for managing OUD, ORT is the most effective and is widely considered the gold standard [33]. ORT has been shown to reduce opioid-related mortality by up to 70% [34-36]. Two formulations are available as ORT in Australia: buprenorphine and methadone [37]. Both of these drugs are selective, long-acting mu-opioid receptor agonists with some differing pharmacodynamic properties; methadone is a full agonist, whilst buprenorphine is a partial agonist with a low dissociation rate with the mu-opioid receptor; however, both bind to the mu-opioid receptor with higher affinity than other opioids, ultimately resulting in long-lasting, protective effects with minimal induction of euphoria [37,38].

The main principle of ORT is to replace non-medical opioid use and manage withdrawal symptoms with long-acting mu-opioid receptor agonists, to elicit anti-craving effects without inducing euphoria [39]. Mechanistically, both methadone and buprenorphine competitively bind to mu-opioid receptors with a higher affinity compared to other opioids, including heroin [38]. This elicits three main effects; withdrawal symptoms are managed and suppressed due to the occupation of mu-opioid receptors; the long-acting effect of these drugs minimises opioid-induced euphoria; and by competitively binding to the mu-opioid receptors, additional concomitant use of non-medical or illicit opioids will have no euphoric effect [22,37,38]. Some physiological side effects from ORT, including gastrointestinal symptoms, can be managed concomitantly with anti-emetics or non-steroidal anti-inflammatory medications to provide symptomatic relief [19]. Eventually, ORTs are progressively tapered to minimise opioid craving and withdrawal symptoms, whilst simultaneously overcoming opiate dependence and withdrawal [8].

Current ORTs have been shown to significantly reduce risky behaviour, crime rates, illicit opioid use, and all-cause mortality since their introduction [6-8]. Despite the clinical success, there are significant limitations with current ORTs, which typically revolve around dependence and development of addiction, relapse, misuse, drug diversion, and poor treatment availability and adherence [10,12,37,40].

Propensity to Cause Dependence and Addiction

As methadone and buprenorphine are opioid agonists, a potential side-effect is the development of iatrogenic dependence and addiction towards ORT. This does pose an ethical debate on the safety and efficacy of using potentially addictive, abusable medications to manage a patient afflicted by a similarly problematic drug [13,40]. As a result, treatment with buprenorphine has increasingly come into favour over methadone, attributable to its pharmacodynamic difference of partial agonism, which decreases the tendency of eliciting euphoria, dependence, and addiction [12,22]. Furthermore, buprenorphine has commonly been co-formulated with the opioid receptor antagonist, naloxone, to reduce euphoric effects and prevent drug diversion [8]. However, despite this safety mechanism, both ORTs can still develop dependence and addiction, cause withdrawal symptoms during the tapering phase, promote relapse, and exacerbate OUDs [10]. Problematically, these withdrawal symptoms can persist for months [26].

Risks During Relapse

Due to the onset of withdrawal symptoms upon ORT tapering, there remains a high risk of relapse. Studies have highlighted that within the first month of ORT discontinuation, the mean relapse rate was approximately 50% [9]. Of note, the consequence of relapse is especially risky after a course of ORT due to the decreased use of opioids from long-term treatment, which leads to a reduction in physiological opioid tolerance, an increase in opioid sensitivity, and subsequently, exacerbated negative effects include respiratory depression, cardiovascular effects, and increased vulnerability of overdose [41,42]. Unsurprisingly, there is an eight-fold increased risk of mortality within the first month immediately after discontinuation of ORT [42].

Misuse of Opioid Replacement Therapy and Drug Diversion

Although the effects of euphoria are mitigated due to the pharmacological long-acting nature of ORTs, there remains a plausible possibility of misuse, particularly for the full mu-opioid receptor agonist methadone [8,12]. Reasons for misuse are varied and can begin with inappropriate and prolonged continuation of ORT due to fear of relapse, or stem from medication abuse for euphoric effects [9]. Certain patient groups with tolerance to opioids, such as those with chronic pain and complex care needs, may require greater opioid doses to achieve adequate levels of analgesia. These populations are important to recognise as judicious opioid stewardship in this group fundamentally differs from those of the opioid-naïve population. Failure to recognise this need can lead to stigmatisation of such populations and poor pain management.

Anecdotally, many individuals who were dissatisfied with their prescribed opioid dose have been described to have sought out opioids in greater amounts, often illicitly or through means of seeking prescriptions from multiple independent prescribed, termed ‘doctor shopping’. An extension of misuse is drug diversion, which is a major public health concern. Drug diversion involves the distribution of methadone or buprenorphine into the black market for illicit recreational purposes with population-level studies highlighting diverted ORTs (methadone in particular) significantly contributing to opioid-related deaths [7,13].

Treatment Availability and Retention

There are a variety of social, economic, and psychological factors that influence ORT availability and retention rates. Studies have estimated that up to 15% of individuals with OUD are on ORT [37,43,44]. Although it is widely used for dependence and addiction, there remains an existing social stigma, preconceived negative views, and a general lack of awareness that ORT is a medically prescribed and evidence-based method to manage OUD [37,40,44]. By extension, some countries (such as Russia) and state systems (such as correctional facilities) discourage or prohibit the use of ORTs [40]. Secondly, the costs of ORTs are high, limiting accessibility [12,26]. Finally, there are strict regulations that govern the prescription and supply of ORT. In Australia, Victorian prescribers must have completed pharmacotherapy training and obtained a permit from the Department of Health for each patient prescribed ORT. Pharmacies too are bound by the regulations that enforce supervised dosing and tight regulations around takeaway doses. Strict regulations around ORT are in the interests of minimising drug diversion but can represent a barrier to entry with potential ORT patients unwilling to frequent their chosen pharmacy location up to seven days of the week to seek treatment due to either inconvenience or stigma. As a result, patients were often involuntarily discontinued from ORT at the discretion of the clinician due to an inability to follow rigorous treatment programs [9]. Hence, despite clinical successes, there is an unmet need for non-addictive, non-opioid therapies in managing OUDs [26].

Cannabidiol

Cannabis, otherwise known as marijuana or *Cannabis sativa*, is a commonly used recreational drug that has been legalised in some countries for recreational and medical indications, including as an analgesic and anxiolytic [33,45]. Constitutively, the main active components of cannabis are cannabinoids, which primarily consist of the non-psychoactive CBD and the psychotomimetic Δ9-tetrahydrocannabinol (THC) [40].

There has been growing interest in the use of medicinal cannabis-related products for the management of OUD. As the legal use of medicinal cannabis becomes more prevalent worldwide, studies have recently highlighted exciting associations between medicinal cannabis use and decreasing opioid abuse, demonstrating its potential in managing OUD. Firstly, implemented policies that allow the legal use of medicinal cannabis with adequate availability in dispensaries have shown lower opioid prescriptions, decreased non-medical opioid abuse, and reduced rates of mortality (from opioid overdose), as shown by several studies investigating opioid-related deaths within many US states between the period of 1999 and 2010 [46-48]. Secondly, many individuals reported that using medicine to self-manage OUDs improved symptoms of opioid withdrawal, anxiety, and gastrointestinal upset [49-51]. Finally, in a large survey (n = 2897), 97% of participants reported that the use of medicinal cannabis-related products for chronic pain management resulted in the reduction of opioid use [52]. A study found that prescription opioid use decreased by 40-60% with medicinal cannabis use, where patients reported greater satisfaction and preference for medicinal cannabis-related products compared to prescription opioids [53]. Although these studies are observational and rely on self-reporting and surveys thus increasing the risk of bias, these results highlight the significant potential of medicinal cannabis-related products in ameliorating the opioid crisis.

Of all the constituents of cannabis, CBD shows the greatest promise for the treatment of OUD [54]. The key advantage of CBD rather than whole cannabis and THC is due to its non-psychoactive properties, which eliminate the risk of dependence and addiction [55]. This is particularly important as individuals affected by OUD are more likely to develop cannabis use disorder [19]. Furthermore, a non-psychoactive agent with existing widespread social acceptance may be more appealing to patients and minimise the psychosocial barriers to entry [56]. The safety profile of CBD is modest and typically elicits only mild side effects, such as diarrhoea and moderate sedation [57]. Additionally, CBD can be safely co-administered with opioids in the event of possible concomitant opioid relapse during treatment [44,58]. CBD has two main avenues of interest in addressing the OUD; CBD has been investigated for its direct potential as a non-opioid alternative for managing opiate addiction, and since there is growing evidence and greater leniency towards implementation of medicinal cannabis as an effective analgesic, CBD has a potential role in pain management that may reduce prescription opioid use and indirectly reduce the incidence of OUD [59,60].

Role in Opioid Dependence and Addiction

CBD has been posited to influence various signalling pathways within the body to manage opioid dependence and addiction. It is a non-competitive negative allosteric modulator of cannabinoid receptor type 1 (CB1R) and part of the endocannabinoid system [19,23]. During opioid abuse, disruptions of dopaminergic signalling triggered by CB1R activation in the endocannabinoid system may further potentiate dopamine release; all of which is hypothesised to be involved in neurological maladaptations resulting in OUD [23]. These include the development of emotional associations to substance-related cues, reward salience, and compulsive substance use habits that eventually result in dependence and addiction [23]. As CBD negatively influences CB1R signalling, it can potentially attenuate dependence and addiction [23,27]. Secondly, CBD is an agonist for serotonin 1A (5-HT1A) receptor, part of the serotonergic pathway. Serotoninergic pathways have been shown to be disrupted with substance use disorders, which are associated with impulsivity, addiction, and relapse [28]. The effects of CBD on the serotonergic system have been shown to reduce craving and relapse, elicit anxiolytic effects, and improve stress management through complex pathways [61]. Finally, CBD is an allosteric modulator of the mu-opioid receptor; part of the opioid signalling pathway [40]. Interestingly, the CB1R receptors are colocalised with the mu-opioid receptors, hence, there is an overlap between the endocannabinoid and opioid signalling systems [19]. CBD action on the mu-opioid receptor has been shown to attenuate opioid withdrawal symptoms [24]. Overall, CBD may exhibit multiple mechanisms for managing opiate dependence and addiction without psychoactive properties, which is conducive to its suitability as a non-opioid therapeutic. Currently, multiple clinical studies have shown that CBD treatment has significant impacts on reducing craving, anxiety, and attention towards environmental cues that trigger drug-seeking behaviour in patients with OUDs [57,62,63]. However, it is important to note that there is a paucity of high-powered randomised control trials and evidence for the use of pure CBD for managing opioid withdrawal symptoms, especially since the current literature on medicinal cannabis is conflicting [64,65]. Whilst it is an exciting new agent in the management of OUD, further research will be required to fully realise the potential of CBD.

Reduction of Prescription Opioid Use

CBD has been postulated to be able to replace or act as an adjunct to opioids as an analgesic; if so, it has the potential to decrease opioid use, thereby indirectly reducing OUD. Despite medicinal cannabis being an established analgesic, pain management with pure CBD has only been reported anecdotally and is currently understudied [26,45,66]. It is also important to note that there are no pure CBD medications approved for pain management [26]. It is posited that CBD can elicit analgesic effects through effects on the endocannabinoid, inflammatory, and nociceptive systems [67]. Firstly, CBD has been shown to activate transient receptor potential cation channel subfamily V member 1 (TRPV1) and transient receptor potential cation channel, subfamily A member 1 (TRPA1) receptors, which subsequently decreases inflammation and reduces the secretion of pro-inflammatory molecules [67]. These anti-inflammatory effects are posited to induce analgesic effects [55,68]. Secondly, CBD action as a positive allosteric modulation of mu-opioid receptors may result in endocannabinoid-opioid system interactions to induce analgesic effects [24]. Current clinical studies have shown conflicting clinical evidence for the effects of cannabinoids in pain management. In one study, topical CBD oil was shown to significantly reduce peripheral neuropathic pain [69]. However, other studies demonstrate that CBD has no significant effect on pain management [70,71]. Additionally, CBD may have the potential as an adjunct treatment for prescription opioids due to endocannabinoid-opioid interactions, which may reduce the amount of opioids required in pain management [53]. In particular, a study demonstrated that combination treatments using CBD and prescription opioids resulted in hyper-additive pain relief, resulting in overall reduced opioid use and side effects [72]. However, it is a novel outlook into the adjunctive role of CBD in pain management as a non-opioid therapeutic option that may reduce prescription opioid use to ameliorate the opioid crisis.

Clinical Considerations

When considering the clinical applications of CBD in OUD, an area that remains poorly characterised is the clinical aspects of dosing and adverse effects. The underlying reason for this is explained by the regulation of these substances, both in research and therapeutical applications, which therefore limits the jurisdictions to which clinical trials can take place, as well as the patients that can access these medications.

With respect to dosing, at current there is no best-practice recommended dosing of CBD for OUD. Studies have shown varying dosages for other indications. For example, in chronic pain, efficacy has been found with respect to doses of 20.8 mg of CBD per day in improving neuropathy. This is, however, confounded by the self-titration of CBD products and their integration with THC, which makes interpreting the dose of CBD as well as its effect quite difficult [73]. In anxiety disorder, clinical trials have found a dose of 600 mg of CBD monotherapy taken 90 minutes prior to public speaking could significantly improve anxiety levels compared to control [73]. Jurisdictions whereby CBD has been approved for medicine use, such as Canada, have shown the efficacious introduction of products like Sativex® spray (THC 2.7 mg: CBD 2.5 mg), which has been approved for neuropathic pain and multiple sclerosis with good efficacy [67]. With respect to OUD, a more recent study by Suzuki et al. evaluated the effects of single-dose CBD at 600 mg in improving cue-induced cravings in patients with OUD already on methadone or buprenorphine [63]. In this study, CBD was shown to reduce drug-related cues and cravings. Despite these initial outcomes, OUD as an indication has yet to be thoroughly explored and therefore the doses of CBD required for efficacious ORT remain poorly characterised with a call for dose-response studies and more rigorous and higher-powered randomised-controlled clinical trials needed to better evaluate this effect to guide OUD policies and frameworks when it comes to considering CBD integration.

Similarly, the adverse effect profile of CBD remains under-explored. Notably, a significant research gap remains in research that characterised the long-term effects of CBD, which is mainly attributed to the fact CBD has only recently become approved for clinical therapeutic and research use and therefore it is expected there is a lag time for robust research with adequate follow-up length to develop. Nonetheless, there is preliminary evidence of the adverse effects associated with cannabinoids (inclusive of THC and CBD), including gastrointestinal side effects (nausea, vomiting, and in the severe form, cannabis-induced hyperemesis syndrome), cognitive impairment and drowsiness, and risk of mental health disorders (including mania, anxiety, and psychosis, among others) [70,73]. With respect to the latter, paradoxically CBD and medicinal cannabis products have also been reported to alleviate such symptoms and conditions. Other studies, however, have shown CBD is well tolerated, with no incidence of any adverse effects [63]. It appears that there may be dose-dependent relationships to explain this; however, these are similarly poorly understood. Overall, the prevalence and incidence of these adverse events remain unclear, further alluding to the need for further research to better characterise the tolerability and safety profile of CBD prior to widespread clinical application.

Current limitations and future directions

Despite the significant potential of medicinal cannabis-related products (specifically CBD) in managing OUD, it is an understudied field with limited clinical evidence. This is attributable to the long-standing classification of cannabis, whereby its illegal status provides a major barrier to cannabis research and clinical trials [56,73]. However, with increasing decriminalisation and medicinal repurposing of cannabis worldwide, there will be greater accessibility and resources to conduct primary research on the role of CBD [54]. This, however, poses to remain a challenge, with regulations existing in many jurisdictions. Specifically, cannabis and by extension medicinal cannabis base products remain listed as a Schedule 1 drug, which continues to impede research efforts and slow the progress of drug development [74]. Fortunately, in 2022, the US passed the “Medical Marijuana and Cannabidiol Research Expansion Act” to streamline the research in this space and a step towards important milestones for the introduction of new drugs, including the generation of robust research to meet the requirements of the US Food and Drug Administration (FDA). Similar challenges have been experienced in other jurisdictions, with bodies such as the Therapeutic Goods Administration (TGA) of Australia limiting medicinal cannabis-based products to be prescribed solely by authorised prescribers and limited licenses being distributed for cultivation, production, and manufacture of cannabis-based medicinal products. Despite these improvements, the products on the market have inconsistent quality and control standards and therefore hinder the development of evidence-based best practice guidelines for clinical applications. In this way, jurisdictions worldwide would benefit similarly from more robust research that addresses questions of dosing and safe stock handling and supports the development of formalised training and safety guidelines.

From the current scope of the literature, there are many promising results from observational studies and clinical trials indicating the potential role of CBD in managing opiate dependence and addiction [57,62,63]. Future studies using high-power randomised-controlled trials (with controlled routes of administration, evaluation of dose, and inclusion of greater sample sizes with more rigorous and standardised measures) will be beneficial in elucidating and generating evidence for the potential of CBD in managing OUD [14,45,73]. Future studies investigating CBD in pain reduction and as an opiate adjunct treatment for pain management would further elucidate the potential of CBD as a non-opioid analgesic, which may reduce opioid use and downstream OUDs.

## Conclusions

Opioid medications, both licit and illicit, have led to significant morbidity and mortality worldwide. Approaches have aimed to improve these outcomes for those with opioid dependence and OUD through ORT. Although shown to be effective, ORTs have significant limitations, one of which is the propensity to induce dependence and addiction. This calls for an unmet need for non-addictive, non-opioid alternatives in managing OUDs. Preliminary and preclinical evidence has found that CBD may target pathways that may improve addiction and dependence; however, the clinical applications of this are challenged by a lack of high-quality and randomised clinical trials as well as government policy regarding medical marijuana products. Additionally, at this point in time, there is a poor understanding of the best-practice considerations for CBD implementation, including an understanding of the efficacious dosages for CBD-based OUD therapy as well as a lack of clearly defined tolerability and side-effect profiles. Our review calls for further robust research prior to the consideration of CBD in informing evidence-based policies and frameworks related to ORT.
